# Effects of Neurally Adjusted Ventilatory Assist on Lung Ultrasound Aeration and Diaphragmatic Function in Extremely Preterm Infants With Evolving or Established Bronchopulmonary Dysplasia

**DOI:** 10.1002/ppul.71885

**Published:** 2026-10-19

**Authors:** Anay Kulkarni, Justin Richards, Touqueer Fatima, Shavindra Chellen, Donovan Duffy, Sandeep Shetty

**Affiliations:** ^1^ Neonatal Intensive Care Centre, St George's University Hospitals NHS Foundation Trust London UK; ^2^ Department of Neonatology, William Harvey Hospital East Kent Hospitals University NHS Foundation Trust Ashford UK; ^3^ City St George's University of London London UK; ^4^ Faculty of Life Sciences and Medicine, King's College London UK

**Keywords:** bronchopulmonary dysplasia, diaphragm ultrasound, extremely preterm, lung ultrasound aeration score, mechanical ventilation, NAVA, neurally adjusted ventilatory assist, patient–ventilator synchrony

## Abstract

**Background:**

Neurally adjusted ventilatory assist (NAVA) delivers ventilatory support proportional to diaphragmatic electrical activity, improving patient–ventilator synchrony. Its effects on lung aeration and diaphragmatic workload in extremely preterm infants have not been well characterized.

**Objectives:**

We hypothesized that initiation of NAVA in preterm infants born at < 32 weeks' gestation would result in improved lung recruitment while allowing effective ventilation at lower airway pressures without increasing diaphragmatic workload.

**Methods:**

This prospective observational pilot study was conducted in a tertiary neonatal intensive care unit. Nineteen preterm infants (median gestational age 24 + 5 weeks; median birth weight 700 g, range 380–1352 g) were transitioned to NAVA at a median postnatal age of 30 + 5 days according to a pre‐specified unit protocol. Lung ultrasound aeration scores (LUS), diaphragmatic excursion and thickness, peak electrical diaphragmatic activity (EAdi peak), mean airway pressure (MAP), and FiO_2_ were measured immediately before and 1 h after NAVA initiation. Paired statistical analyses were performed.

**Results:**

LUS decreased markedly following NAVA initiation (mean 15.21 ± 3.26 vs. 7.11 ± 2.47; *p* < 0.001; Cohen's d = 3.02), with the effect observed in both invasive and non‐invasive NAVA groups. MAP decreased significantly (11.82 ± 1.63 vs. 9.56 ± 1.64 cmH_2_O; *p* < 0.001) and FiO_2_ showed a non‐significant trend towards improvement (0.35 vs. 0.32; *p* = 0.020). Diaphragmatic excursion (7.05 ± 2.1 vs. 6.7 ± 2.2 mm; *p* = 0.650) and EAdi peak (8.4 vs. 8.3 mV; *p* = 0.839) were unchanged.

**Conclusions:**

In this pilot cohort, initiation of NAVA in extremely preterm infants was associated with rapid improvement in lung ultrasound aeration scores and reduced ventilatory pressures, without evidence of increased diaphragmatic loading. These findings are hypothesis‐generating and larger controlled studies are needed to confirm them.

## 1 Introduction

Preterm infants born before 32 weeks' gestation frequently require prolonged invasive or non‐invasive respiratory support due to surfactant deficiency, structural lung immaturity, poor chest wall stability, and immature respiratory control. Despite advances in neonatal ventilation strategies, ventilator‐induced lung injury (VILI) and bronchopulmonary dysplasia (BPD) remain significant contributors to morbidity and long‐term respiratory impairment in this population [1, 2]. Reducing volutrauma and barotrauma while maintaining effective ventilation is therefore a key therapeutic goal.

Conventional ventilatory modes rely on pressure‐ or flow‐based triggering, which may be unreliable in preterm infants due to small tidal volumes, high respiratory rates, air leaks, and variable respiratory drive. Patient–ventilator asynchrony is common and has been associated with increased work of breathing, higher ventilatory pressures, and impaired gas exchange [3].

Neurally adjusted ventilatory assist (NAVA) delivers ventilatory support proportional to the infant's neural respiratory drive, detected via electrical activity of the diaphragm (Edi). By coupling ventilator assistance directly to diaphragmatic activation, NAVA improves synchrony and may allow adequate ventilation at lower airway pressures [4, 5]. While physiological benefits of NAVA have been described, objective evidence demonstrating its effect on lung recruitment in extremely preterm infants remains limited.

Lung ultrasound (LUS) is an increasingly validated bedside tool for assessing lung aeration in neonates, correlating with oxygenation, respiratory severity, and risk of BPD [6, 7, 8]. Diaphragmatic ultrasound provides complementary information on respiratory muscle function and workload. We hypothesized that initiation of NAVA in preterm infants born at < 32 weeks' gestation would result in improved lung recruitment, reflected by improved lung ultrasound aeration scores, while allowing effective ventilation at lower airway pressures without increasing diaphragmatic workload.

## 2 Methods

### 2.1 Study Design, Setting, and Participants

This prospective observational pilot study was conducted in a tertiary‐level neonatal intensive care unit at St George's University Hospitals NHS Foundation Trust, London. Data were collected between January 2024 and September 2025. The study was approved by the Research Ethics Committee, Health Research Authority and Health and Care Research Wales (reference 23/SW/0100) and the Trust Research and Development Department; written informed consent was obtained from parents or legal guardians prior to enrollment. The study was conducted in accordance with the Declaration of Helsinki.

Infants born at < 32 weeks' gestation who required invasive or non‐invasive respiratory support for more than 2 weeks, and for whom the clinical team decided to initiate NAVA, were eligible. The decision to initiate NAVA followed a pre‐specified unit protocol with the following indications: (i) requirement for invasive mechanical ventilation beyond 2 weeks of age; (ii) FiO_2_ > 0.40 on non‐invasive respiratory support; or (iii) evolving or established BPD, including severe BPD, as classified by the Jobe and Bancalari criteria [1]. Infants were excluded if parental consent was not obtained or if ultrasound assessment could not be safely performed due to clinical instability.

Where an infant underwent transition to NAVA on more than one separate clinical occasion (e.g., following re‐intubation after extubation failure), each episode was treated as an independent observation, yielding 22 episodes across 18 infants who completed paired assessment; four infants contributed two episodes each at distinct clinical timepoints. A sensitivity analysis excluding the second episode from these four infants was performed to assess robustness of findings. In all cases, the mode of respiratory support (invasive or non‐invasive) was the same before and after NAVA initiation; thus, each infant was compared with their own baseline on the same support modality.

### 2.2 Ultrasound Assessments

Each infant underwent lung and diaphragmatic ultrasound immediately prior to NAVA initiation and 1 h after initiation of optimized NAVA support, during both invasive and non‐invasive NAVA. Lung ultrasound was performed using a GE Vivid S70N machine with a high‐frequency linear probe by a single operator with > 5 years' experience in neonatologist‐performed lung ultrasound. All scans were acquired and scored by the same operator, who was aware of the timing of each assessment. Intra‐ and inter‐observer reliability were not assessed. Image analysis was performed offline with attempts to minimize recognition of assessment sequence; however, complete blinding to clinical timing was not possible. Lung ultrasound aeration scores were assigned using the validated Brat scoring system [6]: each lung was divided into three regions (upper anterior, lower anterior, lateral), each scored 0–3 based on aeration pattern, giving a total possible score of 0–18, where higher scores indicate worse aeration. Pre‐ and post‐NAVA assessments were performed 1 h apart in the same infant. During this interval, no change in body position and no suctioning were performed. Underlying lung pathology was therefore unchanged between assessments, and each infant served as their own control.

Diaphragmatic ultrasound was performed concurrently using a micro‐convex transducer with the infant supine during quiet, regular breathing. The diaphragm was visualized via a subcostal approach [9, 10]; M‐mode ultrasonography was used to measure diaphragmatic excursion (perpendicular distance between the most cephalic inspiratory and expiratory positions) and diaphragmatic thickness during inspiration (ADIT) and expiration (ADET) at most mobile part of the hemidiaphragm, as previously described in ventilated preterm infants [11]. Three measurements were obtained per hemidiaphragm and averaged. MAP, FiO_2_, and peak electrical diaphragmatic activity (EAdi peak) were recorded from the ventilator display at the time of each assessment.

### 2.3 Statistical Analysis

Paired‐samples *t*‐tests were used for within‐subject comparisons of continuous variables, presented as mean ± standard deviation or median (range) as appropriate. Effect size was calculated using Cohen's d. A *p* < 0.05 was considered statistically significant. Statistical analysis was performed using IBM SPSS Statistics, Version 26 (IBM Corp., Armonk, NY, USA). This was a pilot/exploratory study; no formal sample size calculation was performed as no comparable prior study existed at the time of design. Given the exploratory nature and limited sample size, paired analyses were used; however, mixed‐effects modeling accounting for repeated observations within infants would be preferable in a larger cohort. Where sufficient statistics were available, 95% confidence intervals (CI) for mean differences were calculated.

## 3 Results

Nineteen preterm infants were screened and enrolled. Median gestational age was 24 + 5 weeks (range 23–28 weeks) and median birth weight was 700 g (range 380–1352 g). Median postnatal age at NAVA initiation was 30^+5^ days (range 25 + 4–38 + 3) (Table 1). Of 22 episodes, 10/22 (45%) involved infants on invasive respiratory support at NAVA initiation. One infant did not complete a paired assessment because of clinical instability. The remaining 18 infants contributed 22 NAVA episodes (four infants contributed two episodes each at distinct clinical timepoints). Post‐NAVA assessment could not be completed in 3 episodes due to clinical instability, leaving paired LUS data for 19 episodes and paired diaphragmatic data for 18 episodes. Baseline characteristics of enrolled infants are summarized in Table 1.

**Table 1:** Baseline characteristics of enrolled infants (*n* = 19).

Characteristic	Value
Gestational age, weeks	24 + 5 (23–28)
Birth weight, g	700 (380–1352)^2^
Sex	Male 10 (53%); Female 9 (47%)
Postnatal age at NAVA initiation, days	32 (7–81)
Invasive respiratory support, *n* (%)	12 (63%)
Non‐invasive respiratory support, *n* (%)	7 (37%)
Diagnosis at enrollment	Evolving or established BPD (all)
Total NAVA episodes analysed	22^3^

*Note:* Values are median (range) unless otherwise stated. ^2^Birth weight available for 17/19 infants. ^3^Four infants contributed two NAVA episodes each at distinct clinical timepoints.

Abbreviations: BPD, bronchopulmonary dysplasia; NAVA, neurally adjusted ventilatory assist.

### 3.1 Lung Ultrasound Aeration Score

There was a marked reduction in LUS following NAVA initiation: mean pre‐NAVA 15.21 (SD 3.26) versus post‐NAVA 7.11 (SD 2.47), a mean reduction of 8.1 points (95% CI 6.81–9.39; paired *t* (18) = 13.16, *p* < 0.0001; Cohen's d = 3.02) (Table 2, Figure 1, Image 1, 2). The improvement was observed following both invasive and non‐invasive NAVA initiation, and the sensitivity analysis excluding duplicate episodes yielded consistent results.

**Table 2:** Summary of lung ultrasound aeration scores, diaphragmatic and ventilatory parameters before and after NAVA initiation (*n* = 18–19 paired observations).

Variable	Pre‐NAVA Mean (SD) or Median (range)	Post‐NAVA Mean (SD) or Median (range)	*p* value
Lung ultrasound aeration score (LUS; 0–18)	15.21 (3.26)	7.11 (2.47)	< 0.001*
Invasive NAVA subgroup	15.6 (3.1)	7.4 (2.6)	< 0.001*
Non‐invasive NAVA subgroup	14.8 (3.5)	6.8 (2.3)	< 0.001*
Average diaphragmatic excursion (ADE), mm	7.05 (2.1)	6.7 (2.2)	0.650
Mean airway pressure (MAP), cmH_2_O	11.82 (1.63)	9.56 (1.64)	< 0.001*
FiO_2_	0.35 (0.21–0.60)	0.32 (0.21–0.65)	0.020*
EAdi peak, mV	8.4 (3.2–20.6)	8.3 (4.7–17.1)	0.839
Diaphragmatic inspiratory thickness (ADIT), mm	6.1 (1.9)	5.4 (1.6)	0.110
Diaphragmatic expiratory thickness (ADET), mm	5.1 (1.3)	4.9 (1.4)	0.520

*Note:* Subgroup LUS values estimated from available data. *Statistically significant (*p* < 0.05).

Abbreviations: ADE, average diaphragmatic excursion; ADET, average diaphragmatic expiratory thickness; ADIT, average diaphragmatic inspiratory thickness; EAdi, peak electrical diaphragmatic activity; LUS, lung ultrasound aeration score (Brat scoring system, 0–18; higher = worse aeration); MAP, mean airway pressure; NAVA, neurally adjusted ventilatory assist; NS, not significant; SD, standard deviation.

**Figure 1:**
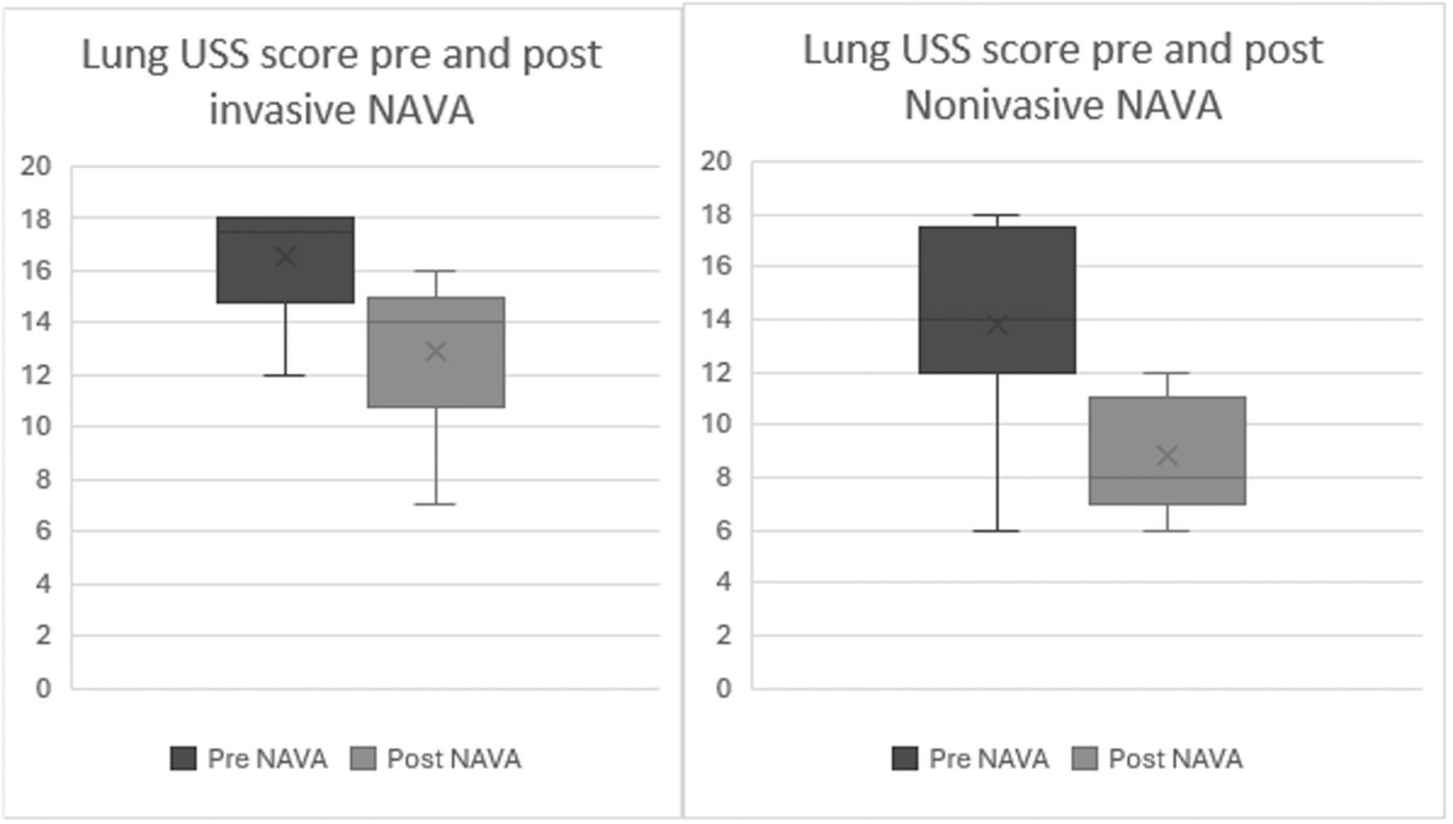
Lung ultrasound aeration scores (LUS) before and after NAVA initiation shown separately for invasive NAVA (left panel) and non‐invasive NAVA (right panel), with individual paired observations and group means.

**Image 1:**
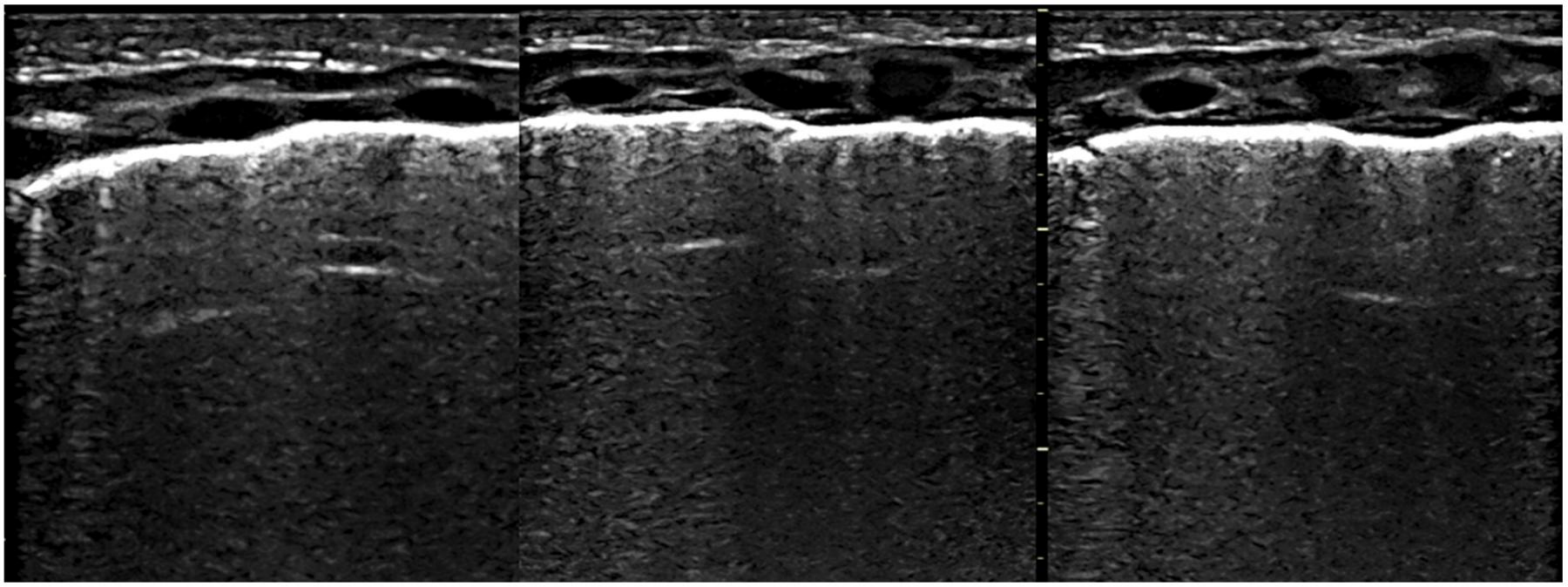
Representative lung ultrasound images before NAVA initiation demonstrating severe loss of aeration with multiple B‐lines, subpleural consolidations, and absent A‐lines, consistent with a high lung ultrasound aeration score.

### 3.2 Ventilatory Parameters

Ventilatory parameters. MAP decreased significantly following NAVA initiation (11.82 ± 1.63 to 9.56 ± 1.64 cmH_2_O; mean reduction 2.26 cmH_2_O (95% CI 1.38–3.14); paired *t*(17) = 5.40, *p* < 0.001). FiO_2_ showed a non‐significant trend towards improvement (0.35 ± 0.10 to 0.32 ± 0.10; *p* = 0.020) (Table 2, Figure 1, 2). Positive end‐expiratory pressure (PEEP) did not change significantly.

**Figure 2:**
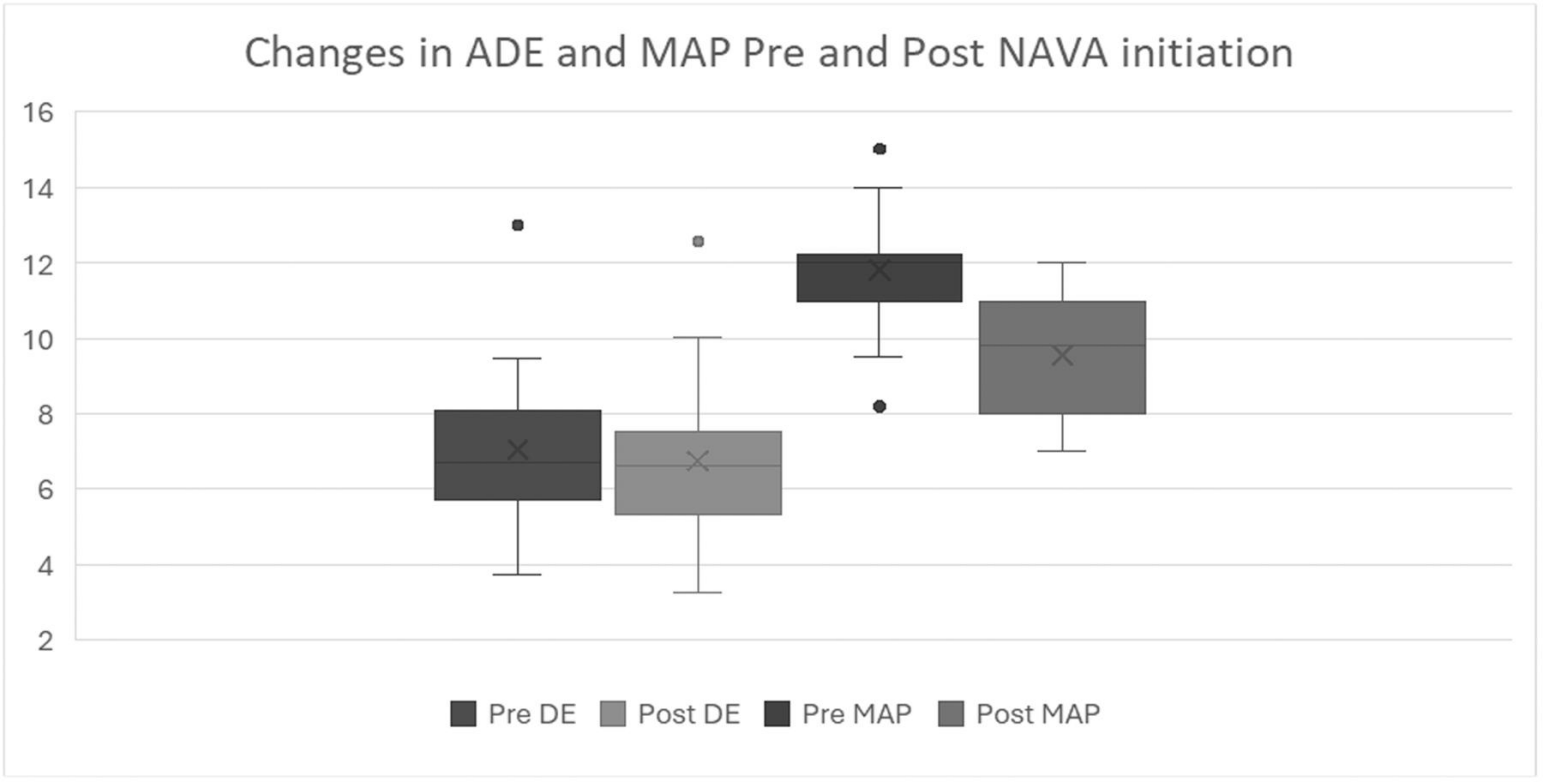
Ventilatory and diaphragmatic parameters before and after NAVA initiation; Left Panel showing Diaphragmatic excursion (DE) and right Panel showing Mean Airway Pressure (MAP).

### 3.3 Diaphragmatic Excursion and Neural Drive

Average diaphragmatic excursion did not change significantly (7.05 ± 2.1 vs. 6.7 ± 2.2 mm; *p* = 0.65). Median EAdi peak was 8.4 mV (range 3.2–20.6) pre‐NAVA and 8.3 mV (range 4.7–17.1) post‐NAVA (*p* = 0.839). Spearman correlation analysis demonstrated a significant positive correlation between EAdi peak and diaphragmatic excursion pre‐NAVA (ρ = 0.73, *p* = 0.001) and post‐NAVA (*ρ* = 0.50, *p* = 0.036). No significant correlation was observed between EAdi peak and LUS aeration score (pre‐NAVA: *ρ* = −0.39, *p* = 0.112; post‐NAVA: *ρ* = 0.16, *p* = 0.523) (Table 2, Image 2).

**Image 2:**
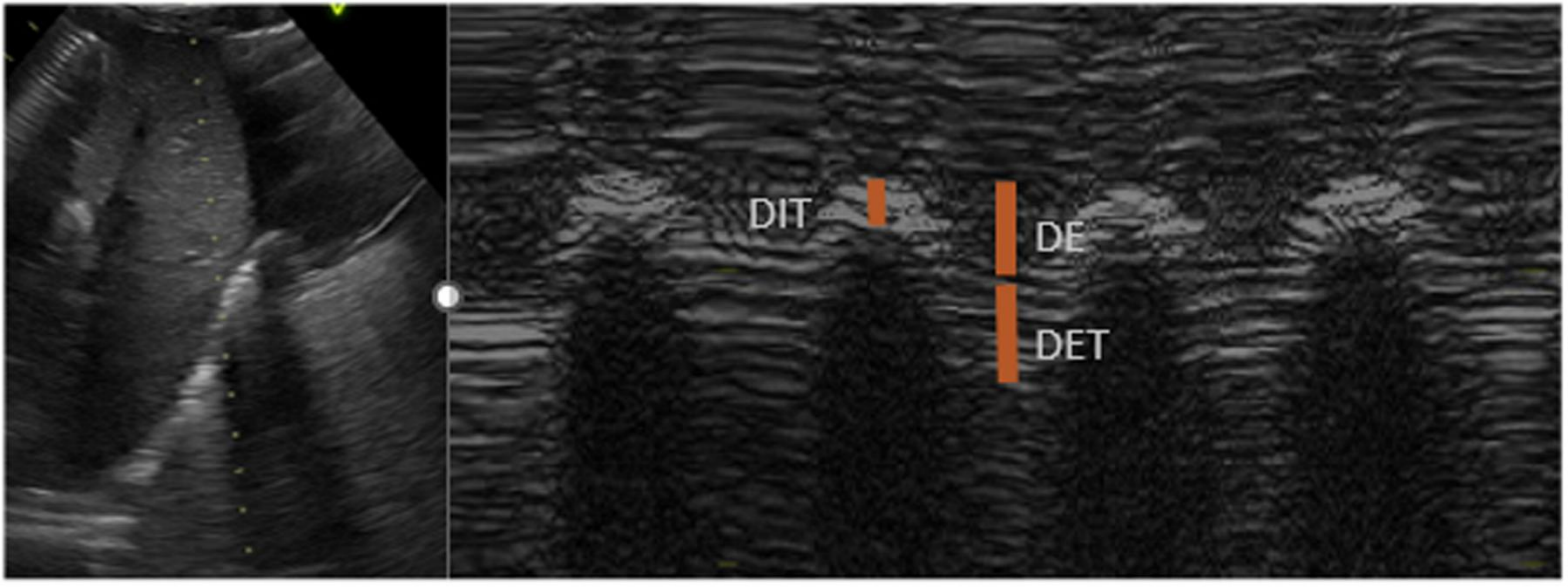
Representative lung ultrasound images and diaphragmatic M‐mode tracings 1 h after NAVA initiation, demonstrating marked improvement in lung aeration with return of A‐lines and reduction in B‐line density, alongside preserved diaphragmatic excursion. [Color figure can be viewed at wileyonlinelibrary.com]

### 3.4 Diaphragmatic Thickness

A non‐significant reduction in both ADIT (6.1 ± 1.9 vs. 5.4 ± 1.6 mm; *p* = 0.110) and ADET (5.1 ± 1.3 vs. 4.9 ± 1.4 mm; *p* = 0.520) was observed following NAVA initiation (Table 2).

## 4 Discussion

This prospective observational study demonstrates that initiation of NAVA in extremely preterm infants is associated with a rapid and substantial improvement in lung ultrasound aeration scores, alongside reduced ventilatory pressures and oxygen requirements, without any evidence of increased diaphragmatic workload. To our knowledge, this is the first study to objectively characterize the effect of NAVA on lung ultrasound aeration scores in this population.

### 4.1 Lung Recruitment and Ventilatory Efficiency

The magnitude of improvement in LUS (mean reduction 8.1 points; Cohen's d = 3.02) is striking. A pre‐NAVA mean score of 15.21 on an 18‐point scale corresponds to severe loss of aeration, consistent with the cohort's clinical profile of evolving or established BPD. LUS has been validated as a surrogate for lung aeration and correlates with oxygenation and respiratory severity in neonates [6, 7, 8]. The rapid improvement within 1 h of NAVA initiation likely reflects enhanced patient–ventilator synchrony, with more consistent tidal ventilation reducing atelectasis in dependent lung regions [3, 4]. This magnitude of change is large and should be interpreted cautiously; observer bias and measurement variability cannot be excluded, and independent replication is needed.

### 4.2 Airway Pressure and Diaphragmatic Preservation

The significant reduction in MAP despite unchanged diaphragmatic excursion and EAdi peak suggests that NAVA allows effective ventilation at lower distending pressures for a given level of diaphragmatic activation. Cumulative volutrauma and barotrauma are strongly implicated in the pathogenesis of VILI and BPD [1, 2]. Previous studies have demonstrated reductions in peak inspiratory pressure with NAVA in neonates [4, 5, 12]; our findings extend this by providing objective, ultrasound‐based evidence that reduced airway pressure is accompanied by genuine improvement in lung aeration rather than deterioration or compensatory respiratory effort. A recent study in extremely preterm infants demonstrated that inspiratory effort and dynamic transpulmonary driving pressure are closely related to diaphragmatic activation [13], supporting the physiological rationale for patient‐triggered ventilatory support such as NAVA.

The absence of significant changes in diaphragmatic excursion, EAdi peak, or thickness suggests that NAVA does not increase respiratory muscle load. This is particularly important in preterm infants, who are vulnerable to diaphragmatic fatigue and disuse atrophy during prolonged ventilation [14, 15]. A prospective observational study comparing NIV‐NAVA with nasal intermittent positive‐pressure ventilation (NIPPV) in preterm infants demonstrated higher diaphragmatic excursion on NIV‐NAVA [16], consistent with our finding of preserved diaphragmatic function following NAVA initiation. The non‐significant trend towards reduced diaphragmatic thickness post‐NAVA should be interpreted with caution; given the small sample size and absence of statistical significance, this likely reflects random variability.

### 4.3 Limitations

This study has several important limitations. The sample size is small and the observational design means the clinical decision to initiate NAVA was not randomized. The small sample limits precision and generalizability, and the study was underpowered to detect smaller changes, particularly in diaphragmatic parameters; non‐significant results should not be interpreted as evidence of no effect. Concurrent clinical changes at the time of NAVA initiation – such as adjustments to diuretics, corticosteroids, or sedation – were not systematically recorded and could have influenced results, particularly the LUS findings; this is an important confound that must be addressed in future work. Four infants contributed two episodes each, and whilst the sensitivity analysis excluding duplicate episodes yielded consistent results, a mixed‐effects modeling approach would be preferable in a larger dataset. All ultrasound scans were acquired and scored by a single operator who was aware of the timing of each assessment, and reliability (intra‐ and inter‐observer) was not assessed. This carries a risk of measurement and observer bias. Future studies should use anonymised scans, independent blinded readers and formal reliability testing. The operator was not fully blinded to clinical context, assessments were limited to 1 h post‐initiation, and long‐term outcomes such as duration of ventilation or BPD severity were not assessed. Objective respiratory mechanics and gas exchange variables, including tidal volume and pCO_2_, were not systematically collected. Stricter anatomical localization of ultrasound views across assessments should be incorporated into future study protocols. Infants in whom ultrasound could not be safely performed because of clinical instability were not included in the paired analysis, so the findings may not apply to the most unstable infants. Although the modality of respiratory support was unchanged within each infant, the invasive and non‐invasive groups may have differed in baseline lung aeration, and the pooled estimate should be interpreted in that context. The baseline median LUS score was higher in the invasive group than the non‐invasive group (17.5 vs. 14.0; *p* = 0.07). Diaphragmatic thickness was measured subcostally with a micro‐convex probe rather than at the zone of apposition [9, 10], an approach used previously in ventilated preterm infants [11]. This may reduce accuracy and comparability with other studies. As the same operator, equipment and technique were used before and after NAVA in each infant, systematic error should have limited effect on within‐infant change, although random error would not cancel. Thickening fraction was not measured.

## 5 Conclusions

Initiation of NAVA in extremely preterm infants is associated with significant improvement in lung ultrasound aeration scores, reduced airway pressures, and lower oxygen requirements without increasing diaphragmatic workload. These findings are hypothesis‐generating and provide early physiological evidence supporting NAVA as a potentially lung‐protective ventilatory strategy. Larger controlled studies, incorporating detailed characterization of concurrent interventions, tidal volume, respiratory rate and pCO_2_ data, and serial ultrasound assessments with standardized imaging protocols, are required to confirm these findings and determine whether improved lung recruitment with NAVA translates into reduced duration of ventilation and lower rates of BPD.

## Author Contributions

**Anay Kulkarni:** conceptualization, investigation, formal analysis, data curation, writing – original draft. **Justin Richards:** writing – review and editing. **Touqueer Fatima:** project administration. **Shavindra Chellen:** project administration, writing – review and editing. **Donovan Duffy:** writing – review and editing. **Sandeep Shetty:** conceptualization, methodology, visualization, validation, writing – review and editing, project administration, supervision.

## Funding

The authors have nothing to report.

## Ethics Statement

The study was approved by the Research Ethics Committee, Health Research Authority and Health and Care Research Wales (reference 23/SW/0100) and the Trust Research and Development Department. Written informed consent was obtained from parents or legal guardians. The study was conducted in accordance with the Declaration of Helsinki.

## Conflicts of Interest

S.S. participated in a one‐off Medical Advisory Board meeting for Maquet Critical Care AB in 2024. He has received honoraria from Getinge for educational speaking engagements related to neonatal ventilation. The other authors declare no conflicts of interest.

## Data Availability

Data sharing not applicable to this article as no datasets were generated or analysed during the current study.
